# Financial Management of Listed Companies Based on Convolutional Neural Network Model in the Context of Epidemic

**DOI:** 10.1155/2022/1871315

**Published:** 2022-09-17

**Authors:** Qian Duan, Xinyu Cao, Li Xu

**Affiliations:** The School of Economics and Management, Cangzhou Jiaotong College, Huanghua 061100, Hebei, China

## Abstract

The goal of financial management is to manage the purchase and sale of assets, the rational financing of funds, the management of cash flow in operations, and finally, the reasonable distribution of company profits in a certain task situation, which is simply the management of the “three statements” of the enterprise. The core issue of the financial mechanism is how to choose a centralized or decentralized management model, which requires the company to consider the internal and external environment, and according to the development of the company, the quality of employees and business characteristics of various factors, in order to make the best choice of the company's financial management model. Therefore, in the context of the epidemic, this article conducts research related to the financial management of listed companies based on convolutional neural network models (radial basis neural network, generalized regression neural network, wavelet neural network, and fuzzy neural network). This article, firstly, discusses the basic theories of macro- and micro-financial management of enterprises and financial management of listed enterprises, secondly, examines the overall financial management model of listed enterprises in China through methods such as the convolutional neural network model research method introduced in this article, and then, after an overall examination and analysis of the financial management situation of X-listed enterprises, finds the macro- and micro-status quo of financial management of listed enterprises in China under the epidemic, and in the sub. On the basis of the status quo, suggestions are made to build a financial management model that combines centralization and decentralization and to build a group financial risk management system.

## 1. Introduction

The goal of financial management is to manage the purchase and sale of assets, the reasonable financing of funds, the management of cash flow in operation, and finally, the reasonable distribution of company profits in a certain task situation, which is simply the management of the “three statements” of the enterprise. Due to the different methods of allocating financial power within the company, the financial model can be divided into “centralized,” “decentralized,” and “mixed.” At present, the centralized financial management model has been commonly used in the world's major companies, according to statistics, more than 80 percent of the world's top 500 companies have established a centralized financial management model. The core issue of financial mechanism is how to choose a centralized or decentralized management model, which requires the company to consider the internal and external environment, and according to the development of the company, the quality of employees and business characteristics and other factors, in order to make the best choice of the company's financial management model [1].

Regardless of the form of organization, exposure to certain risks is inevitable. In the twenty-first century, changes in the technological and economic environment have made organizations pay more attention than ever to the issue of risk. Among the various types of risks, financial risks are more important in the case of enterprises. After all, generally speaking, the main purpose of a company is to make a profit. Group companies are one of the more advanced forms of corporate organization. Once the financial risk of a group of companies goes wrong, it can have a very significant impact not only within this enterprise, but also on the stability of society [2]. The collapse of well-known companies such as Arthur Andersen and Kenmore is closely related to financial management, showing that their impact cannot be underestimated, both in China and abroad. The financial risk management of enterprise groups is also attracting more and more attention and many researchers are studying this issue, trying to prevent and reduce the harm caused by financial risks by increasing the importance of the stakeholders of enterprise groups through the study of financial risk management. The constant cases of financial risk leading to corporate bankruptcy have increased the sense of urgency and the need for theoretical research [3].

Group companies are organizations that have evolved from individual companies and naturally have the general characteristics of individual companies' financial risks, such as speculation, objectivity, and fragmentation. However, it also has some more complex characteristics of its own, such as dynamicity, complexity, and comprehensiveness. Due to these characteristics, it is more difficult to identify, assess, and manage the risks of group companies' financial risks. Since the promulgation and implementation of the CE financial management system (COSO-ERM for short), a lot of research has been conducted and discussed by domestic and foreign researchers, trying to make it more operable and effective [4]. However, any program should be used for us, not simply follow the “fetishism,” our country's enterprises need to improve the actual political and economic environment in China, and can be based on the CE financial management system to establish a framework system for China's enterprises, to build their own financial risk defense system, and ultimately achieve the enterprise's purpose [5].

## 2. Research Background

### 2.1. Research on Enterprise Micro-Financial Risk Management

Chao [6], starting from the analysis of the financial management work mode of corporate enterprises, illustrates that no matter what type of management mode is faced with business risks, the only way to better avoid the occurrence of business risks in enterprise financial management is to follow the management development ideas of flattening hierarchical management, modularization of services, networking of information management, and integration of big data [7]. Professor Gang [8] studied in-depth the basic meaning and principles of risk management of group financial management and gave strategies and measures for prevention and resolution in the face of corporate financial risks [9]. Ouyang [10] studied the main mechanisms of internal financial risk generation in companies and used AVIC as an example to clarify the measures that companies should adopt in their internal financial risk management practices [11]. Professor Pengjie [12] provides an in-depth analysis of the causes of corporate financial risks, while providing a slightly different perspective from other literature research approaches to strengthen corporate risk management, and conducts countermeasures research at various levels such as the strategic financial level, organizational level, internal control level, early warning level, performance level, information and culture of the company, but has not been able to establish a rigorous theoretical framework system [13]. Minhui [14] analyzed the causes of financial risk in a listed company from four major aspects, namely, financing, inputs, capital operation, and profit distribution of the company, and conducted an overall study of its formation characteristics, having specific management policy advice from the perspective of the characteristics of financial risk [15]. Through the above-mentioned literature, we see that most of the studies on corporate financial risks from a microscopic point of view have always been based on theoretical knowledge of the concept, characteristics, and transmission of business risks of corporate financial conditions, and there is a one-sided disadvantage of “treating the head when the head hurts and treating the foot when the foot hurts” in the recommendations for the overall financial management of the enterprise. However, in the context of the company's strategy and development, it is important to consider the overall financial management proposal. However, there are only a few studies that propose specific objectives of enterprise financial risk management based on the company's strategy and development, and the overall objectives of the company according to its financial risk tolerance. It is not in a practical point of view, the establishment of a specific structure of financial enterprise risk management, nor in accordance with the requirements of the enterprise financial risk management objectives, the implementation of the main body of responsibility, improve the procedural approach, improve the protection system, and optimize the management basis [16].

### 2.2. Research on Enterprise Macro-Financial Risk Management

As a new theory, the application of the CEE financial management system in China has led to some problems, such as the lack of awareness of risk management work by the responsible state and the formal construction of risk mechanisms. It is pointed out in his study that CE financial management system policy must be further adjusted if it is to be successfully implemented in the current market economy, but the adjusted policy framework system is not available in his study [18]. The CE financial management system faces the main problem of fixation, that is, the decision-making implementation contains subjective decisions, and there is still a lack of clear theoretical guidance for the architecture, thus requiring the management of the company to comprehend the meaning of the architecture and the main issues in a comprehensive and in-depth manner. On the basis of an in-depth analysis of the shortcomings of the CE financial management system, an all-round risk management architecture with a “dual core” of internal control and risk management control is proposed. However, the new framework and the CE financial management system also have difficulties such as being too theoretical and obstacles to the translation of the guidance into practice [19, 20]. Zinkewicz [21] explored the current status of ERM implementation in the insurance industry, and the effective implementation of ERM will face challenges from various aspects such as system design and data acquisition and integration. Similar results were obtained in a related study by Garcia [22]. The results of another study by Beasle et al. [23] also indicate that there are still some financial barriers to the advancement of ERM [24].

Teng [25] explored the suggestion of establishing an overall architecture for enterprise risk management in China by describing the development of enterprise risk management work on internal control architecture and using enterprise risk management work architecture. She also argues that according to the current level of risk in China, the establishment of an effective Chinese system framework is not yet possible overnight. She also emphasized that emphasizing the central role of directors in the construction of corporate structure, defining corporate risk management objectives, actively exploring and establishing a risk assessment system, and creating a corporate management culture are the keys to the successful establishment of an overall corporate framework system [26]. Based on the CE financial management system architecture, Yang [27] proposed that the establishment of a corporate risk management framework that is beneficial to China should establish a corporate risk management committee, an effective corporate regulatory system, and other specific measures [28]. Taking Shaanxi Electric Power Company as an example, Xiaohan [29] attempted to establish a risk management work structure dedicated to the electric power industry and based on the five elements of internal company management. After the summary of these articles, it is seen that on the one hand, CE financial management system as a basic technical theoretical framework system, which is really implemented to the actual enterprise still needs to do the corresponding technical implementation level of conversion and refinement [30]. However, the reality in China is that in the long-term survival and development of Chinese enterprises, it is necessary to establish their own unique characteristics, which means that if the CE financial management system is directly adopted, it will become an unavoidable reality, which will negatively affect the effectiveness of the framework implementation. On the other hand, the summary of the literature on the establishment of financial risk architecture in other companies shows that such articles are more inclined to establish their own new architecture based on the CE financial management system and supplement the original one accordingly. In the strictest sense, the study of such articles must always take the CE financial management system as the basic analytical starting point of the study, without leaving this research framework to provide a unique risk management system [31].

## 3. Materials and Methods

### 3.1. Basic Theory

#### 3.1.1. Listed Companies

A listed company is a specific component of a joint stock company, which publicly issues shares, reaches a considerable size, and has its shares approved by law to enter the centralized securities trading market for trading. A joint stock limited company applying for the listing and trading of its shares shall submit relevant documents to the stock exchange. The stock exchange decides whether to accept its shares for listing and trading in accordance with the provisions of this Law and relevant laws and administrative regulations [32].

China's securities law makes strict and detailed regulations for listed enterprises, as specified in Article 50. An enterprise applying for an IPO (initial public offering of shares) needs to meet all of the following conditions: (1) the shares have been examined and determined to have been publicly offered by the CSRC; (2) the registered capital of the company has a share capital of not less than RMB 30 million; (3) the publicly offered shares should account for at least 1/4 of the total amount of shares of the company; in large enterprises with total shares of more than RMB 400 million, this ratio can be reduced to 1; (4) the enterprise cannot have major criminal and civil lawsuits as well as illegal and disorderly acts in the last 3 years, and the accounting report has no false records; and (5) the stock exchange can require the actual standard of the enterprise to be higher than the above terms when applying for listing, and report to the State Council for approval and filing. Our listed enterprises must strictly abide by the relevant accounting standards and legal system in China, and must disclose their financial status within the specified period to the market and shareholders in a timely manner. Accounting reports must be disclosed every 6 months during the accounting year. The above conditions greatly reduce the moral hazard and adverse selection of enterprises, can better protect the rights of shareholders, and are the way for China's listed enterprises to improve their quality, expand their scale, and improve their management [33].

#### 3.1.2. Financial Management

Enterprise financial risk management is to evaluate various financial risks identified in advance in the process of achieving the objectives of the enterprise, to understand the possibility and impact of their occurrence, and to propose corresponding solutions in the context of the enterprise's own situation. It is an objective activity to resolve the events that occur in the objective world. At the same time, the enterprise financial risk management is also a dynamic process, as the environment changes, so does the enterprise risk management.

First, we need to understand the basic concepts involved. Risk is understood in a narrow sense and a broader sense. Risk in a narrow sense refers to the loss caused by uncertain future events, while risk in a broader sense is not only about losses, but also about benefits. The company will always face various uncertainties in the process of achieving its goals, so the company's managers must identify risks, so as to assess the possibility of their occurrence and the severity of their impact, and try to manage them effectively to reduce them to a level that the company can bear, and those that cannot be solved can be solved by transferring them. There are many risks in an enterprise, mainly macroeconomic risks such as socio-political risks and natural environment in China, and also micro-risks of the enterprise itself, such as product quality risks and solvency risks. We need to classify the risks of enterprises and propose corresponding risk management approaches in a targeted manner.

At the end of the last century, there were many collapses and failures of large companies in the United States, many of which were due to internal control failures. In order to regulate the internal management of companies, the COSO committee in 1992 developed an integrated framework for internal management, which is the first systematic framework on internal control in the world. The framework was not only recognized by theoretical researchers, but also favored by many companies in practice, and indeed helped companies to avoid possible risks. However, 10 years later, it was found that despite the perfect internal control system, there were cases of risky losses, such as the Enron incident. The SO Act of 2002 required comprehensive risk control for companies, and the COSO Committee of September 2004 was keeping pace with the times by incorporating a company's built-in risk management system based on an integrated structure of internal management controls. The official publication of Enterprise Risk Management – An Integrated Framework reminds the theoretical and practical communities to pay comprehensive attention to risk and strengthen risk management. After the severe financial crisis in the USA in 2008, the COSO committee consulted widely and published an exposure draft of the ERM framework in May 2016 in order to reduce the recurrence of financial crises and to find effective prevention methods, and published the finalized “Enterprise Risk Management-Strategy and Performance Alignment” (ERM 2017). This version has added the integration of strategy and performance management inside, which highlights more the effect of the coordination between strategy, performance and risk management, and has stronger guidance for enterprise risk management, putting strategic risk management into a more realistic business environment to be considered.

### 3.2. Convolutional Neural Network Model Research Method

The following four convolutional neural network models are used in this article: radial basis neural network, generalized regression neural network, wavelet neural network, and fuzzy neural network. Because this aticle involves the financial management of listed enterprises, different research methods are used separately. The models are compared separately and the best model among the four models is simulated.

#### 3.2.1. Radial Basis Neural Network

**Figure 1:**
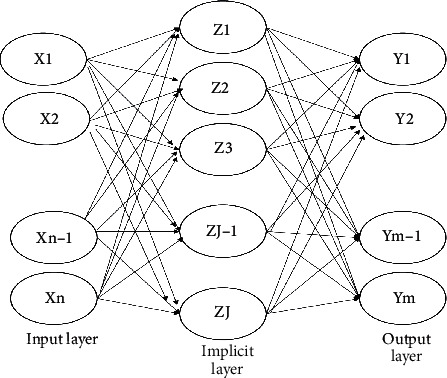
Structure of radial basis neural network.

Radial Basis Function Neural Network (RBFNN) is a most typical three-layer forward neural network structure. In addition to having the information processing of traditional neural networks, its implicit layer uses radial basis functions for nonlinear mapping of input data, which is then passed to the next layer after linear computation. The structure of the radial basis neural network is shown in Figure 1.

In the unsupervised learning part, the data are clustered by using a clustering algorithm such as K-means to obtain the centroid of the radial basis function in the hidden layer, and then the width vector of the radial basis function is calculated by using the centroid information, and the width vector is calculated by the following formula (1).

(1) $$\begin{array}{c} \sigma_{j}=\frac{c_{xy}}{\sqrt{2h}}\text{,} \end{array}$$

where *c*_*Xy*_ is the maximum distance before the centroid and *h* is the number of nodes.

After that, the input data are related to the scattering through the implicit layer and the output layer, respectively, and the output *x*_*i*_ of the first node *j* of the input sample in the implicit layer is calculated by the following equation (2).

(2) $$\begin{array}{c} \phi \left(x_{i},j\right)=\exp\;\left(-\frac{1}{2\sigma_{j}^{2}}x_{i}-c_{i}\right)\text{,} \end{array}$$

where *c*_*j*_ and *σ*_*j*_ are the centroid and width *m* vector of the first node in the hidden layer, respectively.

The output of *x*_*i*_ the first node of *j* the input sample in the output layer is calculated by the following equation (3).

(3) $$\begin{array}{c} y_{m}=\varphi \left(\phi \left(x_{i},j\right)*w_{m}\right)\text{,} \end{array}$$

where *w*_*m*_ is the node weight and *φ* is the activation function.

In the supervised learning part, it is mainly the process of continuously correcting the parameters in each layer, and this process is mainly calculated by the error function to calculate the gradient value of each parameter, and then the parameters are continuously corrected using traditional gradient descent methods such as stochastic gradient descent (SGD), taking the weights used for linear calculation in the output layer as an example, the update formula is as follows (4).

(4) $$\begin{array}{c} w_{t}=w_{t-1}-\frac{u*\sigma E}{\sigma w_{t-1}}\text{,} \end{array}$$

where *E* is the error function and *u* is the learning rate.

In addition to the above methods, the centroids and width vectors of the hidden layer can be directly generated randomly, after which they are updated according to the gradient correction formula of the supervised learning process.

#### 3.2.2. Generalized Regression Neural Network

**Figure 2:**
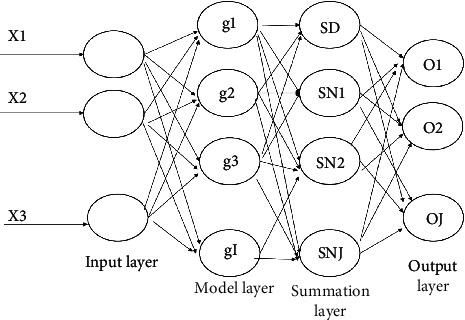
Structure of generalized regression neural network.

Generalized Regression Neural Network (GRNN) is a four-layer forward propagation neural network with fewer parameters and better nonlinear mapping capability, and the data are input to the network and then passed through the input layer, pattern layer, summation layer, and output layer to obtain the output results. The network does not have a training process, but mainly optimizes the smoothing factor of the pattern layer to obtain good output results as shown in Figure 2.

The computational process is not shown in detail here, and the specific computational process can be obtained by inversion of radial basis neural network, which is not done in this case. Although GRNN does not require network training, the smoothing factor of the pattern layer has a large impact on the performance of the network, and too large or too small a smoothing factor will lead to underfitting and overfitting of the network, respectively, and it is usually difficult to set the smoothing factor to a better value in the experiment, so if you want to get better network performance, you generally choose an efficient intelligent optimization algorithm to find the optimal smoothing factor.

#### 3.2.3. Wavelet Neural Network

**Figure 3:**
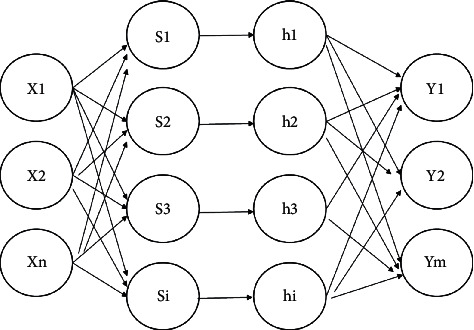
Wavelet neural network structure.

Wavelet Neural Network (WNN) has a three-layer structure, which is characterized by the use of wavelet basis function as the activation function of the neurons in the hidden layer, which makes the network more capable of self-learning when processing data sets with large amounts of data, so it can fit complex relational data faster. Its structure is shown in Figure 3.

The computational process is not shown in detail here, and the specific computational process can be obtained by the radial basis neural network inversion, which will not be done in this example. There are four main parameters in WNN, the size of these four parameter values will directly affect the performance of the network, so the training process of WNN as RBFNN mainly uses the traditional gradient descent method such as stochastic gradient descent (SGD) to continuously correct these four parameters.

#### 3.2.4. Fuzzy Neural Network

**Figure 4:**
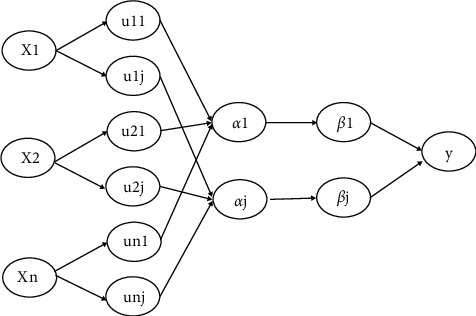
Fuzzy neural network.

Fuzzy Neural Network (FNN) incorporates fuzzy theory into the information transfer process of the network, which can have a larger processing range and faster information processing speed when processing information, so the self-learning ability and mapping of the network is relatively high. The structural diagram of a fuzzy neural network is more commonly used, and can be found in general textbooks as shown in Figure 4.

The data is trained by this neural network in a total of five layers. The first layer is the input layer, where the number of nodes is related to the feature dimension of the data, that is, when the feature dimension of the data is *n*, the number of nodes in the input layer is *n*. Then the data is passed from the input layer to the affiliation function calculation layer, where the affiliation function is used to calculate the affiliation of each node, each node represents an affiliation function, and the number of nodes in this layer is the number of possible fuzzy conditions of the input variables. When the dimensionality of the output variables increases, the weights will be adjusted accordingly. In addition, FNN, like the previous RBFNN and WNN, generally uses traditional gradient descent methods such as stochastic gradient descent (SGD) to optimize the centroids of the affiliation function, the width vector, and the connection weights of the output layer.

## 4. Results and Discussion

### 4.1. Comparison of Financial Management Status Before and After the Epidemic

**Figure 5:**
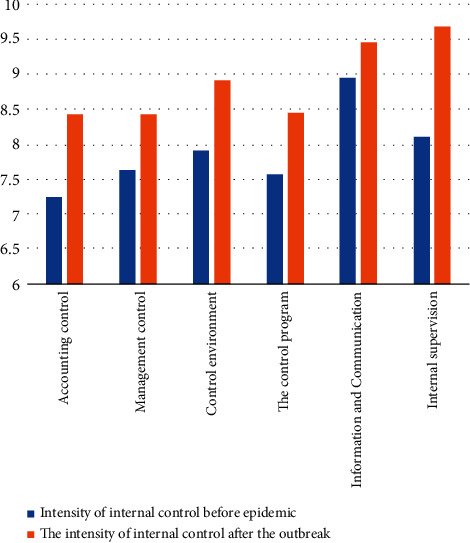
Comparison of the intensity of six major aspects of internal control over financial risk in enterprises before and after the epidemic.

First, the intensity of each of the six major aspects of a firm's internal controls has been strengthened under the epidemic. The strength of each aspect generally ranged from 7 to 10, with higher indicating a greater intensity of control in that aspect of the enterprise. The history of the development of internal control is seen. Swa [34] first studied the essential attributes of internal control in a company, Swa another Ze pointed out that internal control is an integral part of the accounting system and that the company's requirements for internal control originate from within the company. Domestic research scholars also consider the internal management of a company as an internal control system, in which internal control embodies the relationship of authorization and responsibility, and is the way and means to achieve the goals. Both domestic and foreign scholars have summarized it from the perspective of management science and discussed the essential attributes of internal management from the practical needs of the enterprise, instead of just staying in the internal control practice itself. The Ministry of Finance of the People's Republic of China issued “Understanding the audited entity and its environment and assessing the risk of material misstatement” on April 29, 2016, and emphasized that internal management is the design and implementation of policies and procedures by the management and personnel of the audited entity to reasonably determine the authenticity of the audited entity's financial statements, the efficiency and effectiveness of operations, and to reflect the effective implementation of rules and regulations within the audited entity. Policies and procedures. The change in the intensity of corporate control under the epidemic is shown in Figure 5.

**Figure 6:**
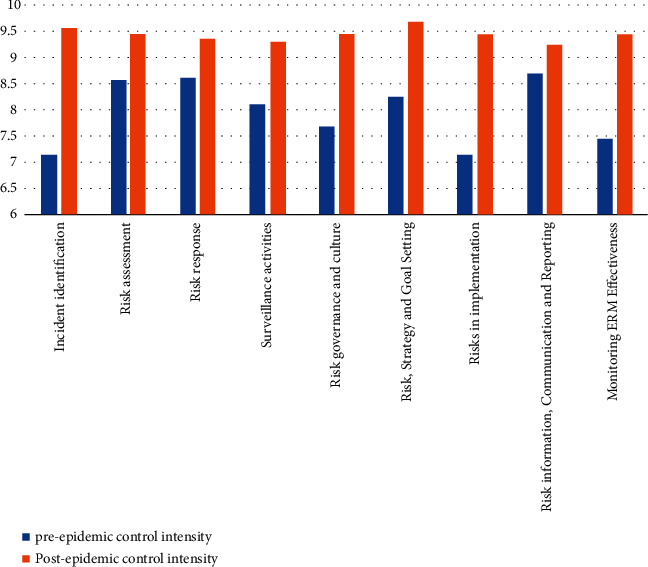
Comparison of the strength of ten secondary aspects of internal control over financial risk in enterprises before and after the epidemic.

Second, the intensity of each of the 10 secondary aspects of corporate internal control was strengthened under the epidemic. The intensity of each aspect generally ranged from 7 to 10, with higher indicating a greater intensity of control in that aspect of the enterprise. It was only in the late 1990s that scholars began to refer back to the “internal control conclusion theory” and the “three elements.” The previous understanding of internal control was rather homogeneous, without effective subdivision, and the role of internal control could not be realized. As the socio-economic environment has changed, the understanding of internal control has deepened, and the basic elements of internal control have changed from three to five: management environment, risk assessment, internal management activities, information dissemination and communication, and internal oversight. Accounting systems are also no longer presented separately and have become information and communication. Control procedures have also been adjusted somewhat, with more refined controls and the formation of risk assessment, control activities, and internal oversight. The changes in these elements are not accidental and reflect the requirements of our new economic environment for companies that can only keep up with the times to help them gain a competitive advantage. The technology-oriented period of internal control changed to management-oriented internal control, several organizations and institutions have not stopped studying the elements of internal control, even if the same organization is constantly developing and improving internal control. As you can see in the chart, we went from 5 elements to 8 elements and later to 5 elements, which is not only a change in numbers, but also a qualitative change in people's understanding of internal control. Exactly what is right and what is wrong is not really conclusive here. We say that the right one is the best one, and the research that can help companies to reduce risks is the most effective one. People's understanding of the nature of internal control varies from era to era; different environments also directly affect the understanding of internal control; and different organizations reach different conclusions about internal control. Different companies will also use different internal control tools and approaches. We can only bring internal control to life by placing it in a specific context as shown in Figure 6.

### 4.2. Analysis of the Current Situation of Financial Management in a Listed Enterprise

After more than 10 years' development, it has established many production bases at home and abroad and expanded the scale of production and sales of soft plastic materials to 11 times, which is the leading level of the same industry at home and abroad in terms of product technology and equipment level, product production and sales scale, and its adaptability to domestic and foreign markets. It is now a leading company of new polymer soft plastic materials and a high-tech company in China. However, the development of a company is not smooth sailing. With the rapid development of the domestic flexible plastic packaging materials industry, as well as the concentration of new production capacity emissions, making the domestic oversupply situation. And as the international crude oil prices continue to soar, driving the company's production of raw materials products rose sharply, product profit margins gradually reduced to the occurrence of losses. At the same time, the downward adjustment of export tax rebate rate and the continuous rise of RMB have gradually reduced the cost advantage of products in the global market. A company's gearing ratio is high and unreasonably composed because of the rapid financing in the early stage. As the interest rate continues to rise under China's macro-tight monetary policy, the company is burdened with risks. Another enterprise's capital chain breaks and the corresponding creditor bank pursues the joint and several guarantee liability of the major shareholder of an enterprise due to the mutual guarantee relationship between the major shareholder of an enterprise and another enterprise, which causes a big panic in the financial industry and will soon spread to an enterprise or the corresponding SMEs with mutual guarantee relationship, making an enterprise's financial risk arise. The main trigger for the explosion of a company's financial risk came from accidental external causes, but it is clear that a company of this size is not just facing financial difficulties due to a several hundred million dollar guarantee crisis. Next, in the future, this article analyzes the causes of a firm's financial risk management failure and its risk treatment measures after the financial risks emerged. In the following, we refer to the research enterprise in this article as this enterprise or enterprise X.

Regarding the choice of financing methods, there are usually debt financing and equity financing, and the company needs to choose the appropriate financing method and financing ratio according to the development stage in which it is located. For this company, the risk in the early stage of development of a new project is high. Choosing a financing method with lower financial risk must keep the overall risk within a certain range. At this time, share-based financing is usually used, and as the project becomes larger and larger, the proportion of debt gradually increases and debt-based financing is used. Now the enterprise is mainly financed by absorbing short-term borrowings and investments from investors. The possession of equity-type capital is large and growing fast, and the enterprise does not need to take big financing risks.

**Figure 7:**
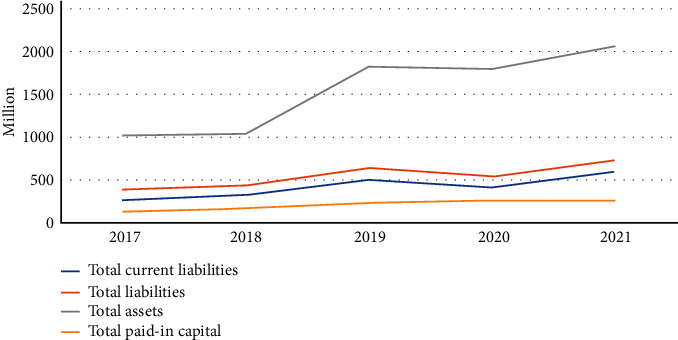
Capital composition of enterprise X from 2017 to 2021.

However, as seen in the graph, the growth rate of the total debt of the company in 5 years from 2017 to 2021 has increased by 2.79%. The growth rate of the total share price is 90.27%. The rate of increase in the company's debt and the rate of increase in the share price are the same. The corporate gearing ratio has been below 50% for the last 5 years. It has been very low. The capital structure of the enterprise remains relatively stable and low. By 2021, the ratio of industrial debt capital to equity assets of enterprises has increased by 35% and by 1.61%, still below 50%, but enterprises are still under considerable debt pressure. Most of the main financing for SMEs is the growth of loans and capital investment from investors, and a certain amount of fixed assets of SMEs are supplied to enterprises. There is a certain financial leverage effect and the repayment pressure of SMEs increases if the gearing ratio is high. The investor's capital investment is conducive to rapid expansion and development of the enterprise, low repayment pressure, but high financing costs. The enterprise has a large asset ratio and a small proportion of liabilities, so there is no greater repayment pressure, but low long-term debt is not good. Overall the capital structure of this enterprise is not reasonable as shown in Figure 7.

**Figure 8:**
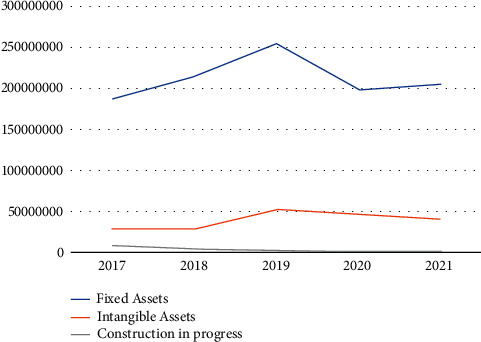
Analysis of investment in each asset of enterprise X from 2017 to 2021.

Enterprise X monitors the execution of the project in real time during the financing process and reflects the progress of implementation and problematic points to the company. The company keeps detailed records of where each amount of funds used goes, and management should keep timely records to avoid exceeding the budget and being out of reality and internal improper events confirming these records to ensure the degree of use of funds and smooth project implementation. Company X is still very strict in the process management, but for the outside of the company, the company did not analyze the impact of risk in detail. The amount of the company's long-term equity investments decreases year by year. The year 2017 was the highest value. It was the first loss-making year since the company was founded, in which the company over-invested and the company took a larger financial risk that year. Investments in pursuit of steady growth: in terms of investment income, the company's foreign investment income is considerable, and after reflecting on the investment failure in 2017, the company pays attention to the investment effect, and the company is now in a more stable investment position than before. The external investments are gradually reduced and turned into investments within the company. The ratio of each part of assets is measured to avoid the accumulation of internal assets due to over-investment, which leads to insufficient operating funds as shown in Figure 8.

As can be seen from the Figure, the investment in various assets of enterprise X from 2017 to 2021, by observing the total amount of these three investments will increase from $43325340.80 in 2017 to $4932194595.30 in 2021, with a slow increase every year, in 2019 is the highest value, and in 2020 is still slowly increasing after a slight decrease, and in 2018 the highest increase, but relatively low. The increase in the size of fixed assets and intangible assets in construction works of Company X originates from the expansion of subsidiaries, and the enterprise invests a lot of money to purchase network resources in order to network projects with the goal of better development. The level of equipment, backwardness within the enterprise, and equipment and production lines that cannot be replaced by generations are eliminated.

### 4.3. Countermeasures for Improvement of Enterprise Financial Risk Management

#### 4.3.1. Build a Financial Management Model that Combines Centralization and Decentralization

The relationship between the enterprise and each subgroup is an asset bond relationship, and from the perspective of capital relationship, the key content of the group's management of the subgroup enterprise company is the effective control of capital and financial activities. “Hierarchical management” means that the group company implements first-level management to each subgroup company, while the subgroup company implements second-level management to the subordinate enterprise group according to the principle of four unified and separate. The core of first-level management is supervision and control, and the core of two-level management is cost management and cash flow. The four unifications, that is, the coordination and unification of information, mechanism, capital and team. Information unification, unified enterprise financial software at all levels, unified financial reporting caliber, and relevant data. It is required to form one account and one set of tables when aggregated within the group to meet the requirements of internal control of enterprises and control of syndicates. The system is unified, and the financial management system, tax policies, accounting methods, and financial software of major enterprise companies are unified and formulated by the group, while local group companies may establish their own implementation rules according to the group's regulations, which must be reported and approved before implementation. The group also conducts inspections on the implementation status of the financial system of each subsidiary. The enterprise capital management is unified, and the entire enterprise company adopts the supervision platform of direct connection between banks and enterprises, and establishes the cash pool system of four subsidiaries to centralize the management and monitoring of all liquid funds of each enterprise company. The centralized monitoring and effective operation of the capital management system, an account to manage the whole enterprise. The team unifies the financial management organization of the small and medium-sized companies under the group as the management organization of the group's business. Each company enterprise has unified the financial setting method and staffing of each enterprise. The financial staff of the group companies and their subordinate enterprise companies are assigned and escorted by the group enterprises, and vertical leadership is implemented. The salaries, rewards, and treatment of the financial and accounting staff are unified and standardized by the enterprises, while the training, recruitment, job transfer, title evaluation, and appointment and removal of the financial staff of the enterprises are unified to the enterprises for arrangement. By the government assigned to the financial organs, in the form of agreements in a variety of personnel management agreements with enterprises or affiliated service companies limited, in order to determine the business content, staff positions, management work standards, and assessment methods. All financial accountants have adopted a job rotation system, in principle, a two-year rotation. “A separation” is the separation of accounting. The products and business results of each enterprise company operate freely in the area of autonomous activities in accordance with the law, with separate cost accounting and self-accounting.

#### 4.3.2. Construction of the Group's Financial Risk Management System

*(1) The Principle of Construction*. According to the management principle of “centralized control and hierarchical implementation,” a capital supervision system with the group's cash asset pool as the core content is established, and the control mode of “hierarchical squeezing, industry segmentation, information system networking and financial data integration” is highlighted in the construction of the group's financial risk control. The control platform should support different forms of financial management control and capital operation within the company in all aspects, and develop and design different levels and modules adapted to the different functions of various forms of financial management institutions, and each level and module can be applied independently of each other, as well as seamlessly integrated and unified.

*(2) Optimize the Financial Organization Structure*. From the perspective of capital relationship, the focus of the group's enterprise management of subgroups is on the effective control of capital and financial activities. Based on the principle of hierarchy and stratification, risks are set for each group according to the strategic view of risk management. The Management Department has rebuilt the organizational structure by setting up an Early Warning Department and a Crisis Management Department under each department. “Hierarchical management” is implemented by group companies for each subgroup company. Hierarchical management is the second level of management by the subgroup companies over the enterprise groups of the subordinate companies based on the principle of four uniform classifications. The core work of the first level of management is supervision and control. The core work of the second level of management is cost management and capital flow. The products and business results of each enterprise company operate freely in the area of autonomous activities in accordance with the law, with separate cost accounting and self-responsibility for profits.

## 5. Conclusion

The financial model can be divided into “centralized,” “decentralized,” and “hybrid” due to the different methods of allocating financial power within the company. The core issue of the financial mechanism is how to choose a centralized or decentralized management model, which requires the company to consider the internal and external environment and to make the best choice of the financial management model based on various factors such as the company's development, staff quality, and business characteristics. Therefore, in the context of the epidemic, this article conducts research related to the financial management of listed companies based on the convolutional neural network model. In this article, firstly, the basic theories of macro- and micro-financial management of enterprises and financial management of listed enterprises are discussed, secondly, the overall financial management model of listed enterprises in China is examined through methods such as the convolutional neural network model research method introduced in this article, and then, after an overall examination and analysis of the financial management situation of X-listed enterprises, the macro- and micro-status quo of financial management of listed enterprises in China under the epidemic is found, and the following suggestions are made on the sub status quo, the following suggestions are made.

1. Construct a financial management model that combines centralization and decentralization. The relationship between enterprises and subgroups is asset bond relationship, and from the perspective of capital relationship, the key content of the group's management of subgroup enterprise companies is the effective control of capital, financial and other activities. “Hierarchical management” means that the group company implements first-level management to each subgroup company, while the subgroup company implements second-level management to the subordinate enterprise group according to the principle of four unified and separate. The core of first-level management is supervision and control, and the core of two-level management is cost management and cash flow. The four unifications, namely the coordination and unification of information, mechanism, capital, and team.
2. Construct the group's financial risk management system. The principles to be followed for the construction. In accordance with the management principle of “centralized control and graded execution,” we establish a capital supervision system with the cash asset pool of the group company as the core content, and highlight the control mode of “hierarchical squeezing, industry segmentation, information system networking and financial data integration” in the construction of financial risk control of the group company. The control platform should support different forms of financial management control and capital operation within the company in all aspects, and develop and design different levels and modules adapted to the different functions of various forms of financial management organizations, and each level and module can be applied independently of each other, as well as seamlessly integrated and unified.

## Data Availability

The dataset can be accessed upon request.
